# PARP Inhibitors Effectively Reduce MAPK Inhibitor Resistant Melanoma Cell Growth and Synergize with MAPK Inhibitors through a Synthetic Lethal Interaction *In Vitro* and *In Vivo*

**DOI:** 10.1158/2767-9764.CRC-23-0101

**Published:** 2023-09-05

**Authors:** Lisa Marie Fröhlich, Heike Niessner, Birgit Sauer, Sofie Kämereit, Eftychia Chatziioannou, Simon Riel, Tobias Sinnberg, Birgit Schittek

**Affiliations:** 1 Division of Dermatooncology, Department of Dermatology, University of Tübingen, Tübingen, Germany.; 2 Department of Dermatology, University of Tübingen, Tübingen, Germany.; 3 Department of Dermatology, Venereology and Allergology, Charité Universitätsmedizin Berlin, Berlin, Germany.; 4 Cluster of Excellence iFIT (EXC 2180) “Image-Guided and Functionally Instructed Tumor Therapies,” University of Tübingen, Tübingen, Germany.

## Abstract

The efficacy of targeting the MAPK signaling pathway in patients with melanoma is limited by the rapid development of resistance mechanisms that result in disease relapse. In this article, we focus on targeting the DNA repair pathway as an antimelanoma therapy, especially in MAPK inhibitor resistant melanoma cells using PARP inhibitors. We found that MAPK inhibitor resistant melanoma cells are particularly sensitive to PARP inhibitor treatment due to a lower basal expression of the DNA damage sensor ataxia-telangiectasia mutated (ATM). As a consequence, MAPK inhibitor resistant melanoma cells have decreased homologous recombination repair activity leading to a reduced repair of double-strand breaks caused by the PARP inhibitors. We validated the clinical relevance of our findings by ATM expression analysis in biopsies from patients with melanoma before and after development of resistance to MAPK inhibitors. Furthermore, we show that inhibition of the MAPK pathway induces a homologous recombination repair deficient phenotype in melanoma cells irrespective of their MAPK inhibitor sensitivity status. MAPK inhibition results in a synthetic lethal interaction of a combinatorial treatment with PARP inhibitors, which significantly reduces melanoma cell growth *in vitro* and *in vivo*. In conclusion, this study shows that PARP inhibitor treatment is a valuable therapy option for patients with melanoma, either as a single treatment or as a combination with MAPK inhibitors depending on ATM expression.

**Significance:**

We show that MAPK inhibitor resistant melanoma cells exhibit low ATM expression increasing their sensitivity toward PARP inhibitors and that a combination of MAPK/PARP inhibitors act synthetically lethal in melanoma cells. Our study shows that PARP inhibitor treatment is a valuable therapy option for patients with melanoma, either as a single treatment or as a combination with MAPK inhibitors depending on ATM expression, which could serve as a novel biomarker for treatment response.

## Introduction

Melanoma cells typically have a high mutational load due to accumulation of unrepaired UV radiation–associated DNA damage (1). Therefore, efficient repair of DNA damage is of fundamental importance for genomic integrity and play a critical role in cancer development and progression. Interestingly, cancer cells are more susceptible to inhibition of DNA damage repair (DDR) compared with noncancerous cells making this pathway an attractive target in cancer therapy especially for patients who suffer from acquired resistance toward targeted drugs (2). Up to 50% of patients with melanoma have a genetic mutation in the *BRAF* gene leading to a hyperactivation of the MAPK signaling pathway and thus benefit from therapy with MAPK inhibitors (MAPKi; ref. 3). However, long-term treatment results in acquired resistance to MAPKi, which limits the benefit of the therapy (4). Interestingly, it still remains unknown whether melanoma cells resistant to MAPKi therapy can be targeted by modulating the DDR.

PARPs are essential in the immediate response to DNA damage. PARP is involved in early steps of the base excision repair, and its inhibition via PARP inhibitors (PARPi) results in the inability to repair the damaged base. In addition, PARPi prevent the dissociation of PARP from damaged DNA leading to PARP trapping and thereby double-strand break (DSB) formation, which is mainly responsible for the cytotoxic effect of PARPi (5). A synthetic lethal interaction of PARPi in tumors with a genetic mutation in homologous recombination repair (HRR) genes makes these patients particularly sensitive to PARPi (6, 7). On the basis of this knowledge, several clinical trials are on the way using PARPi in patients with cancer with a HRR deficiency (HRD) including patients with melanoma (8–10).

Interestingly, inhibition of the MAPK pathway in treatment-naïve melanoma cells was recently shown by us and others to suppress the expression of HRR pathway genes leading to a HRD phenotype (11, 12). This suggests that combined treatment of melanoma cells with PARPi and MAPKi is effective in killing these cells and first preliminary investigations showed a synergistic reduction of cancer cell viability of PARPi in combination with MAPKi (12, 13). However, the molecular mechanisms of synergistic killing of PARPi and MAPKi in treatment-naïve melanoma cells and the effectiveness of PARPi as a monotherapy or in combination with MAPKi in melanoma cells with acquired resistance toward MAPKi is still unknown.

The aim of this study was to decipher whether PARPi therapy can be an appropriate treatment strategy for patients with melanoma and to identify the molecular mechanisms of PARPi sensitivity. In addition, we wanted to unravel whether there are differences in PARPi sensitivity in melanoma cells with an acquired resistance toward MAPKi in comparison with treatment-naïve cells. Finally, we aimed to analyze a potential synergistic effect of PARPi with MAPKi in melanoma cells which are either sensitive or resistant toward MAPKi treatment. Our aim was to unravel which patients with melanoma specifically can benefit from PARPi treatment alone or in combination with MAPKi and to identify biomarkers for treatment response.

## Materials and Methods

### Cell Culture

The human metastatic melanoma cell lines A375, SKMel28, and HT-144 were purchased from ATCC. The human patient-derived xenograft (PDX) cells were received from T. Sinnberg and H. Niessner. The remaining cells were received from M. Herlyn (Wistar Institute, Philadelphia, PA). The BRAF inhibitor (BRAFi) and MEK inhibitor (MEKi) resistant cells were created as described previously (14). BRAFi resistant (R) cell lines were cultured with 2 μmol/L vemurafenib, BRAFi plus MEKi resistant (RR) cell lines were cultured with 2 μmol/L vemurafenib and 5 nmol/L trametinib. All cell lines were cultured in RPMI1640 medium containing 10% FBS (Sigma-Aldrich F9665) and 1% penicillin and streptomycin (Gibco 11548876) at 37°C and 5% CO_2_. The cell lines were regularly tested for *Mycoplasma* using the Venor GeM Classic *Mycoplasma* Detection Kit (Minerva Biolabs 11-1025; last test: November 2022) and used no longer than 2 months upon thawing of the frozen stock. Cell lines were authenticated by short tandem repeat profile analysis (Microsynth; last authentication: November 2022). Primary human fibroblasts were isolated from human foreskin after routinely circumcision from the Loretto Clinic Tübingen upon informed consent of the patients as described previously (15).

### Viability Analysis and Inhibitors

Cell viability was analyzed with the 4-Methylumbelliferyl heptanoate (MUH) assay as described previously (16). A total of 1 × 10^3^ melanoma cells or 5 × 10^3^ fibroblasts were seeded the day before treatment started. Cells were treated with the following inhibitors: vemurafenib (BRAF inhibitor, Medchemexpress HY-12057), trametinib (MEK inhibitor, Biomol 16292-250), LY3009120 (panRAF inhibitor, Medchemexpress HY-12558), olaparib (PARP inhibitor, Medchemexpress HY-10162), or talazoparib (PARP inhibitor, Medchemexpress HY-16106). Experiments were performed in quintuplicates.

### Cell-cycle Analysis

A total of 2 × 10^5^ melanoma cells per cavity of a 6-well plate were seeded 24 hours before treatment start with 2 μmol/L talazoparib for the indicated timepoints. Cells were permeabilized in 80% cold ethanol for 1 hour and resuspended in PBS containing 100 μg/mL RNAseA (Applichem) and 50 μg/mL Propidium Iodide (Sigma-Aldrich P4864) for 30 minutes. LSRII FACS (BCD Biosciences) was used for the cell-cycle experiment. For cell-cycle sorting, cells were treated with 5 μmol/L vemurafenib or remained untreated. A total of 24 hours after the treatment start, cells were harvested and stained with Hoechst (1:20,000, Thermo Fisher Scientific H3570) for 10 minutes and then sorted according to their cell-cycle phase in G_1_-phase, S-phase, and G_2_–M-phase. For the sorting of the cells, FACS Aria (BD Biosciences) was used. FACSDiva software (BD Biosciences) was used for the analysis of the distribution of the cells in the different cell-cycle phases. FloJo_10 was used to visualize the data.

### Apoptosis Assay

Apoptosis assay was performed with the Incucyte Caspase-3/7 Dye for apoptosis (Sartorius 4440) according to the manufacturer's protocol. Briefly, 5 to 10 × 10^3^ cells were seeded into 96-well plate cavities. A total of 24 hours after seeding, cells were treated with 5 μmol/L talazoparib, or remained untreated. Simultaneously, the Caspase-3/7 dye diluted 1:1,000 in cell culture medium was added to the cells. Using the Incucyte SX1, phase contrast and green channels were selected and nine images per well were taken to cover the entire well with an average scan interval of 2 hours for a total of 48 hours. Green fluorescence positive cells were quantified.

### Spheroid Assay

Spheroid assay was performed as described previously (11). For this, 250 cells in 25 μL medium were grown in hanging drops for 10 days. The formed spheroids were then reseeded on a 12-well plate in medium containing 1.2 mg/mL collagen I (Matrix Biosciences, 50105). Spheroids were treated on day 0 with 5 μmol/L talazoparib, 2.5 μmol/L vemurafenib, a combination of both drugs, or remained untreated. Between 5 and 15 spheroids per treatment group were included in the analysis. Spheroid sizes were quantified using ImageJ.

### Colony Formation Assay

Colony formation assay was performed as described previously (16). A total of 750 cells were seeded and 24 hours later treated with 2 μmol/L talazoparib, 2 μmol/L vemurafenib, a combination of both drugs, or remained untreated. Seven days after treatment start, cells were fixed in 4% formalin, stained with 3% crystal violet solution (Sigma-Aldrich, HT90132) in 80% methanol for 2 hours, and colonies were counted.

### RNA Isolation and qRT-PCR

RNA isolation was performed with the help of the Nucleospin RNA Kit (Macherey-Nagel 740955) according to the manufacturer's protocol. RNA isolation of mouse tumor material was performed with NucleoSpin totalRNA formalin-fixed paraffin-embedded (FFPE; Macherey-Nagel 740969) according to the manufacturer's protocol. The cDNA was produced and qRT-PCR was performed as described previously (14). qRT-PCR was performed with the Lightcycler 96 Instrument (Roche). The primer sequences are depicted in Table 1.

**TABLE 1:** Primers used in this project

Gene name	Forward sequence	Reverse sequence
*CDKN1A*	TCACTGTCTTGTACCCTTGTGC	GGCGTTTGGAGTGGTAGAAA
*ATM*	TGTTCCAGGACACGAAGGGAGA	CAGGGTTCTCAGCACTATGGGA
*BRCA1*	TTGTTGATGTGGAGGAGCAA	GATTCCAGGTAAGGGGTTCC
*BRCA2*	GAAAATCAAGAAAAATCCTTAAAGGCT	GTAATCGGCTCTAAAGAAACATGATG
*RAD51*	GGTGAAGGAAAGGCCATGTA	GGGTCTGGTGGTCTGTGTT
*EXO1*	TCGGATCTCCTAGCTTTTGGCTG	AGCTGTCTGCACATTCCTAGCC
*Actin*	CACCATTGGCAATGAGCGGTTC	AGGTCTTTGCGGATGTCCACGT

Actin served as a housekeeping gene. PCR profiler array was performed as described previously (11). RT^2^ Profiler PCR Array Human DNA Repair and p53 Signaling Pathway (Qiagen PAHS-042) were used. Cells were treated with 5 μmol/L talazoparib for 24 hours or remained untreated. Experiments were performed in triplicates.

### Comet Assay

The Comet Assay Kit (Abcam, ab238545) was used for analysis of DNA damage and performed according to the manufacturer's protocol. A total of 1,500 cells were seeded on each cavity of the 3-well object slide. The cells were treated with 15 μmol/L talazoparib, 5 μmol/L vemurafenib, a combination of both drugs, or remained untreated. TBE electrophoresis was performed at 2 V/cm for 20 minutes. For the analysis of the comets, the software OpenComet was used (17, 18). The tail moment was calculated by using the formula: tail moment = tail length × tail DNA %, where the tail length is defined as the length of the tail in pixels and the tail DNA % is defined as the tail DNA content as a percentage of comet DNA content.

### Migration and Invasion Assay

Boyden chamber-based migration and matrigel invasion assay was performed as described previously (19). A total of 8 × 10^5^ cells were seeded onto the transwell insert and 10 minutes after the seeding process, cells were treated with talazoparib, or remained untreated. Experiments were performed in triplicates.

### IHC and Immunofluorescence Staining

FFPE tissue sections of metastatic melanoma samples were deparaffinized and prepared as described previously (11). For IHC staining, ataxia-telangiectasia mutated (ATM) antibody (ab32420, 1:50) was used with Fast Red Substrate (Thermo Fisher Scientific Lab Vision Liquid Fast-Red Substrate System), and a counterstain with hematoxylin and eosin was performed. For the analysis of patient material, written informed consent from the patients was obtained. The studies were conducted in accordance with the Declaration of Helsinki and approved by the Ethics Commission Tübingen, Germany (approval number 866/2021B02). For immunofluorescence staining of ATM, 5 × 10^4^ cells were seeded into each well of an 8-well chamber slide (Falcon 354118). On the next day, cells were fixed with 4% formalin for 10 minutes. The following primary antibodies were used for immunofluorescence staining: anti-ATM (ab32420, 1:250), anti-pH2AX (Ser139; Merck, JBW301, 1:500), anti-p21 Waf1/Cip1 (DCS60; CST2946, 1:50). After overnight incubation of the first antibody at 4°C, the secondary antibodies were added for 1 hour at room temperature. For ATM staining, the secondary antibody Cy3 Donkey Anti-Rabbit IgG (711-166-152, Jackson ImmunoResearch, 1:250), and for pH2AX and p21 the Alexa Fluor 488 Donkey Anti-Mouse IgG (715-546-151, Jackson ImmunoResearch, 1:250) was used. DAPI staining (Invitrogen, NucBlue fixed cell stain Ready-Probes reagent) or Hoechst33342 (Invitrogen) were used to stain the nuclei. Quantification of the IHC was performed by counting the cells with the help of ImageJ. Quantification of the immunofluorescence was performed with the Zen blue Version 2.6 software. For this, a total of 10 images with on average 25 cells per image for each group were analyzed. The red fluorescence intensity per cell was analyzed.

### Immunoblot Assay

Immunoblot analysis was performed as described previously (16). For studying the PARP trapping effect of the PARPi, isolation of the nuclear soluble and chromatin bound fraction was performed using the Subcellular Protein Fractionation Kit for Cultured Cells (Thermo Fisher Scientific 78840) according to the manufacturer's protocol. Cells were therefore treated with 0.005% Methyl methane sulfonate (MMS, Thermo Fisher Scientific, 156890050) and 2 μmol/L talazoparib. The following primary antibodies were used for immunoblot analysis: anti-p21 Waf1/Cip1 (DCS60; Cell Signaling Technology 2946, 1:1,000), anti-pH2AX (Ser139; Merck, JBW301, 1:500), anti-β-Actin (Cell Signaling Technology 4967, 1:2,000), anti-p53 (DOI; sc-126, 1:500), anti-ATM (D2E2; Cell Signaling Technology 2873, 1:1,000), anti-PARP (Cell Signaling Technology 9542, 1:1,000), and anti-H2A.X (Cell Signaling Technology 2595, 1:1,000). The following secondary antibodies were used: anti-mouse IgG, horseradish peroxidase (HRP)-linked (Cell Signaling Technology 7076) and anti-rabbit IgG, HRP-linked (Cell Signaling Technology 7074).

### Xenograft Mouse Experiment

The animal experiments were approved and performed in compliance with the requirements of the German Animal Welfare Act and approved by local authorities (Regierungspräsidium Tübingen, HT1-18). The mice were randomized into the different treatment groups based on gender and age. A total of 1 × 10^6^ melanoma cells (A375 S, A375 R) in 50 μL sterile PBS (Sigma-Aldrich D8537) and 50 μL Matrigel (Corning 354234) were subcutaneously injected into the right flank of NSG mice. The inhibitors talazoparib and vemurafenib were dissolved in 10% DMSO (PanReac AppliChem A3672), 40% PEG300 (Medchemexpress HY-Y0873), 5% Tween80 (Sigma-Aldrich P1754), and 45% PBS. After a palpable tumor size of 25 mm^3^, the treatment with 2 mg/kg/day talazoparib (A375 R), or 1 mg/kg/day talazoparib (A375 S), 25 mg/kg/day vemurafenib (A375 S), or a combination of 1 mg/kg/day talazoparib and 25 mg/kg/day vemurafenib (A375 S) via oral gavage started. The control mice received the solvent without inhibitors. The mice were treated daily for 14 days. The tumor was measured every second day. After 14 days, if the tumor exceeded a size of 1,000 m^3^, or the tumor ulcerated, the mice were euthanized, and the tumor was removed for further analysis. The tumor volume was calculated with the formula: *V*_tumor_ = 0.5 × width^2^ × length.

### Statistical Analysis

All experiments were statistically analyzed using GraphPad Prism version 9.1.2. Data that are statistically significant (*P* < 0.05) were labeled with asterisks (*, *P* < 0.05; **, *P* < 0.01; ***, *P* < 0.001; and ****, *P* < 0.0001). Unless otherwise stated, statistical analysis was performed by unpaired *t* test when two groups were compared with each other, and one-way ANOVA was performed when multiple groups were compared with each other. For the normalized intensity of ATM levels of patients with melanoma before and after MAPK inhibitor resistance, the GSE data provided by Gene Expression Omnibus (GEO; GSE50509 and GSE61992) were used (20–23). Paired Wilcoxon test was performed for the statistical analysis of the GEO data. For the analysis of the MUH cell viability assay, nonlinear regression analysis was performed. When comparing two MUH curves with each other, comparison of fits of the nonlinear regression was performed. Synergism analysis was performed by calculating the combined index (CI) values of each treatment ratio and drug concentration using the software compusyn (https://www.combosyn.com/; with CI < 1 synergistic, CI ≥ 1 additive). In detail, the observed effect of each single treatment and the combined treatment were used. The compusyn software then calculated the CI value using the measured treatment effect of the single treatments versus the combined treatment. The compusyn software calculated the CI value for each treatment ratio used. The calculated CI values were then added in a graphic in respect of the treatment ratio. Protein expression of immunoblot experiments was analyzed with ImageJ 1.53a.

### Data Availability

The data generated in this study are available upon request from the corresponding author. ATM mRNA expression profile data analyzed in this study were obtained from GEO at GSE50509 and GSE61992.

## Results

### BRAFi Resistant Melanoma Cells are Highly Susceptible to PARPi Treatment

**FIGURE 1:**
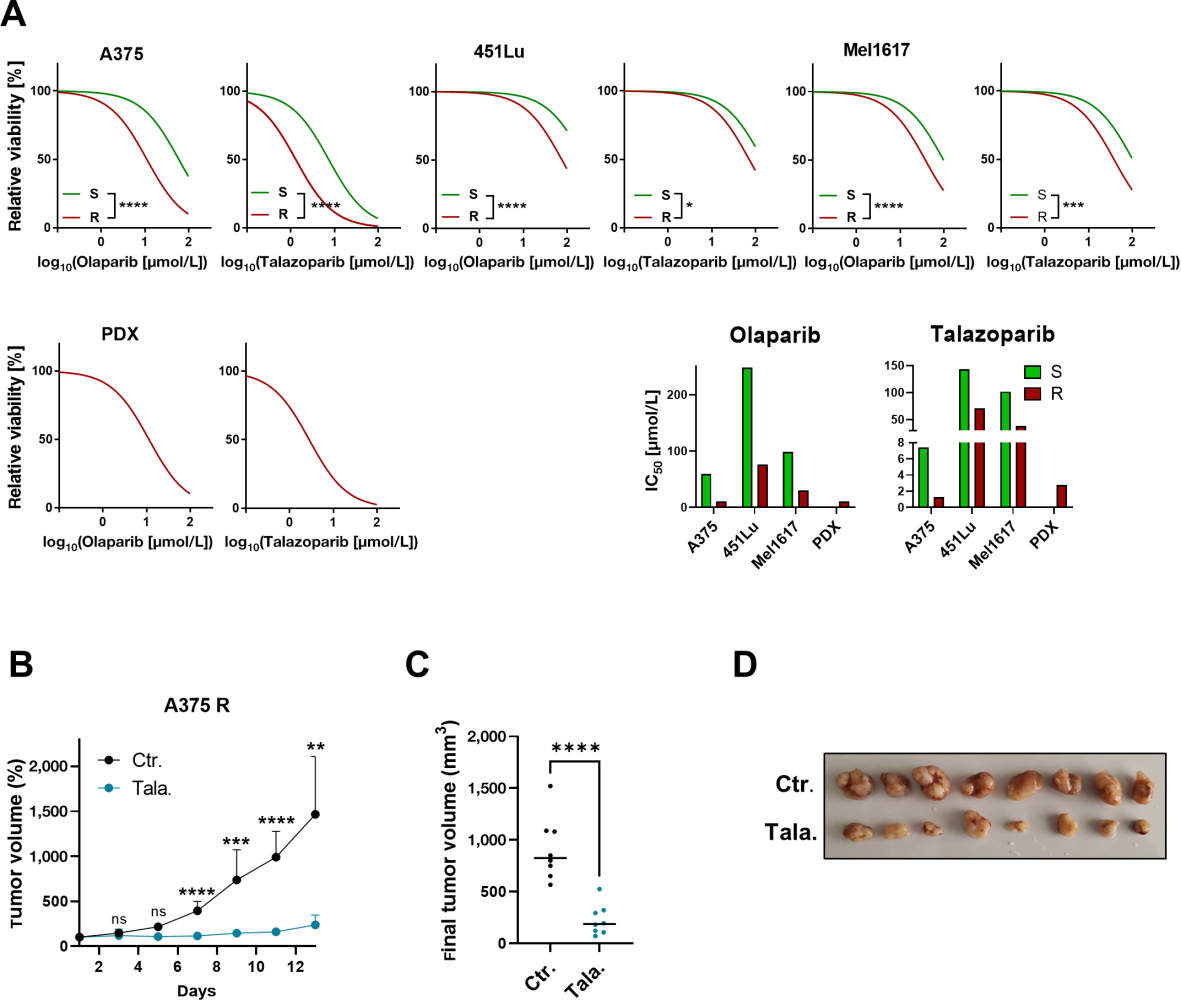
MAPKi resistant melanoma cells are highly susceptible to PARPi treatment. **A,** MUH cell viability assay of vemurafenib sensitive (S) and resistant (R) melanoma cell lines as well as PDX R cells treated with different concentrations of PARPi (olaparib and talazoparib) starting at 100 μmol/L for 72 hours. Comparison of fits of the nonlinear regression was performed to analyze statistical differences between S and R cells. IC_50_ levels are shown. **B,** NSG mice were treated with 2 mg/kg/day talazoparib (Tala.) or the same solvent without talazoparib (Ctr.). The xenograft tumor volume in percent compared with treatment start over time is shown. **C,** The final tumor volume of the individual mice is depicted. Multiple *t* test was used to compare the control (Ctr.) group to the talazoparib (Tala.) group. **D,** Representative pictures of the excised tumors are illustrated.

To analyze whether PARPi treatment affects acquired resistance to MAPKi, we tested the sensitivity of the PARPi olaparib and talazoparib in melanoma cell line pairs that are either sensitive (S) or resistant (R) toward the BRAFi vemurafenib. Interestingly, the R cells were significantly more sensitive to both PARPi (Fig. 1A). Next, we tested the sensitivity to PARPi of a PDX from a patient with melanoma who developed resistance toward the BRAFi vemurafenib and dabrafenib. The cells of this patient were highly sensitive toward the PARPi olaparib and talazoparib (Fig. 1A). Most importantly, treatment with talazoparib almost completely prevented growth of human A375 R melanoma cells in NSG mice. The tumor volume over time of mice treated with talazoparib was significantly reduced from day 7 on compared with the solvent-treated control mice (Fig. 1B). The final tumor size of the mice treated with talazoparib was also significantly lower compared with the solvent-treated control mice (Fig. 1C). In addition, the tumors at the end of the experiment in the talazoparib-treated mice were macroscopically smaller than the solvent-treated control mice (Fig. 1D). These data indicate that BRAFi resistant melanoma cells can be treated with PARPi as a single agent.

### PARPi Treatment Induces p53-associated Proapoptotic Genes and Cell Death in BRAFi Resistant Melanoma Cells

**FIGURE 2:**
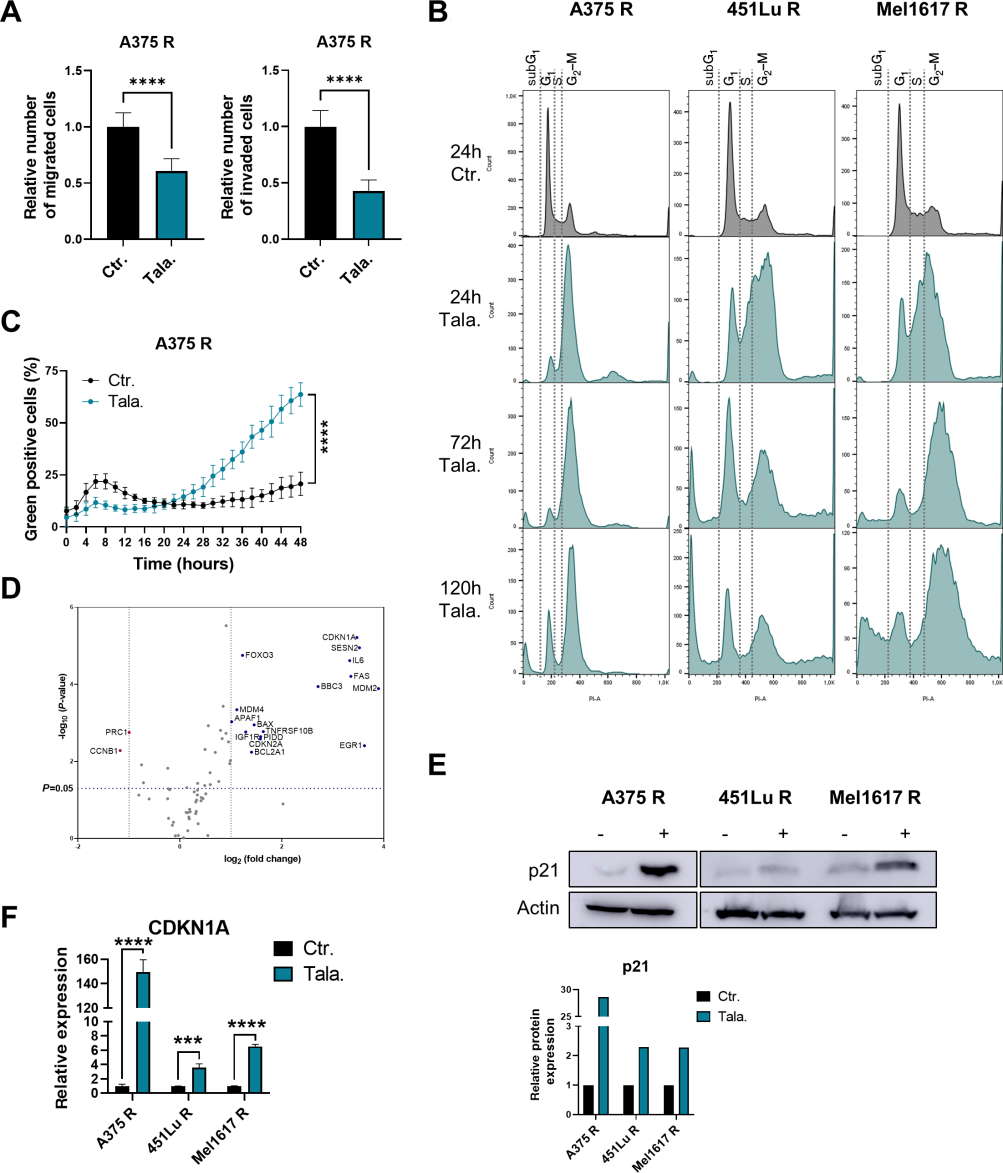
PARPi treatment induces p53-associated proapoptotic genes and cell death in BRAFi resistant melanoma cells. **A,** Migration and Invasion assay using A375 R cells that were treated with 5 μmol/L talazoparib (Tala.) for 24 hours or remained untreated (Ctr.) is shown. The relative number of migrated (left) or invaded (right) cells is shown. Unpaired *t* test was performed to compare the Ctr. with Tala. group. **B,** Cell-cycle analysis was performed in A375 R, 451Lu R, and Mel1617 R cells after treatment with 2 μmol/L talazoparib (Tala.) for 24, 72, or 120 hours. Untreated cells (Ctr.) served as a control. **C,** Incucyte Caspase-3/7 Dye for apoptosis was used to trace cell death activation after treatment of 5 μmol/L talazoparib (Tala.) for up to 48 hours compared with untreated cells (Ctr.). The percentage of green fluorescence positive cells is shown. Unpaired *t* test was performed to compare the Ctr. with Tala. group. **D,** RT^2^ Profiler PCR Array Human p53 Signaling Pathway was performed in A375 R cells that were treated with 5 μmol/L talazoparib for 24 hours or remained untreated. log_2_ fold changed in the expression of the corresponding genes between the talazoparib treated group and the control group as well as the log_10_*P* values of the respective gene expression changes are shown in the volcano plot. Technical triplicates were performed. **E,** Immunoblot analysis of A375 R, 451Lu R, and Mel1617 R cells treated with 5 μmol/L talazoparib (+) for 24 hours or untreated (−). Relative protein expression is shown. **F,** Relative gene expression of CDKN1A after 5 μmol/L talazoparib (Tala.) treatment for 24 hours or untreated A375 R, 451Lu R, and Mel1617 R cells. Unpaired *t* test was performed to compare the Ctr. with Tala. group.

To decipher the molecular mechanism underlying the high sensitivity of BRAFi R cells toward PARPi, we tested their effect on melanoma progression and cell-cycle arrest induction. Interestingly, treatment of A375 R cells with the PARPi talazoparib for 24 hours significantly decreased their migratory and invasive ability (Fig. 2A). Furthermore, cell-cycle analysis revealed an initial induction of G_2_–M arrest that transited to apoptosis, as seen by the appearance of a subG_1_ peak in talazoparib-treated BRAFi R cells (Fig. 2B). Apoptosis induction by talazoparib was confirmed by measuring the amount of activated caspase 3/7 over time (Fig. 2C). As apoptosis induction is often accompanied by activation of the p53 signaling pathway, we performed a PCR profiler array for genes involved in the p53 signaling pathway. Indeed, genes important in p53-mediated cell-cycle arrest and apoptosis induction, such as FOXO3, SESN2, BAX, APAF1, CDKN1A, MDM2, and CDKN2A were upregulated after talazoparib treatment of A375 R cells (Fig. 2D; Supplementary Table S1). In particular, both RNA and protein expression of p21/CDKN1A were significantly induced in talazoparib-treated BRAFi R cells (Fig. 2E and F). This PARPi-dependent upregulation of p21/CDKN1A was also confirmed in the talazoparib-treated mouse tumors (Supplementary Fig. S1A). These data suggest that PARPi treatment induces G_2_–M arrest and subsequent activation of apoptosis by p53-associated induction of proapoptotic genes.

### MAPKi Resistant Melanoma Cells Exhibit Low Expression of the DNA DSB Sensor ATM

**FIGURE 3:**
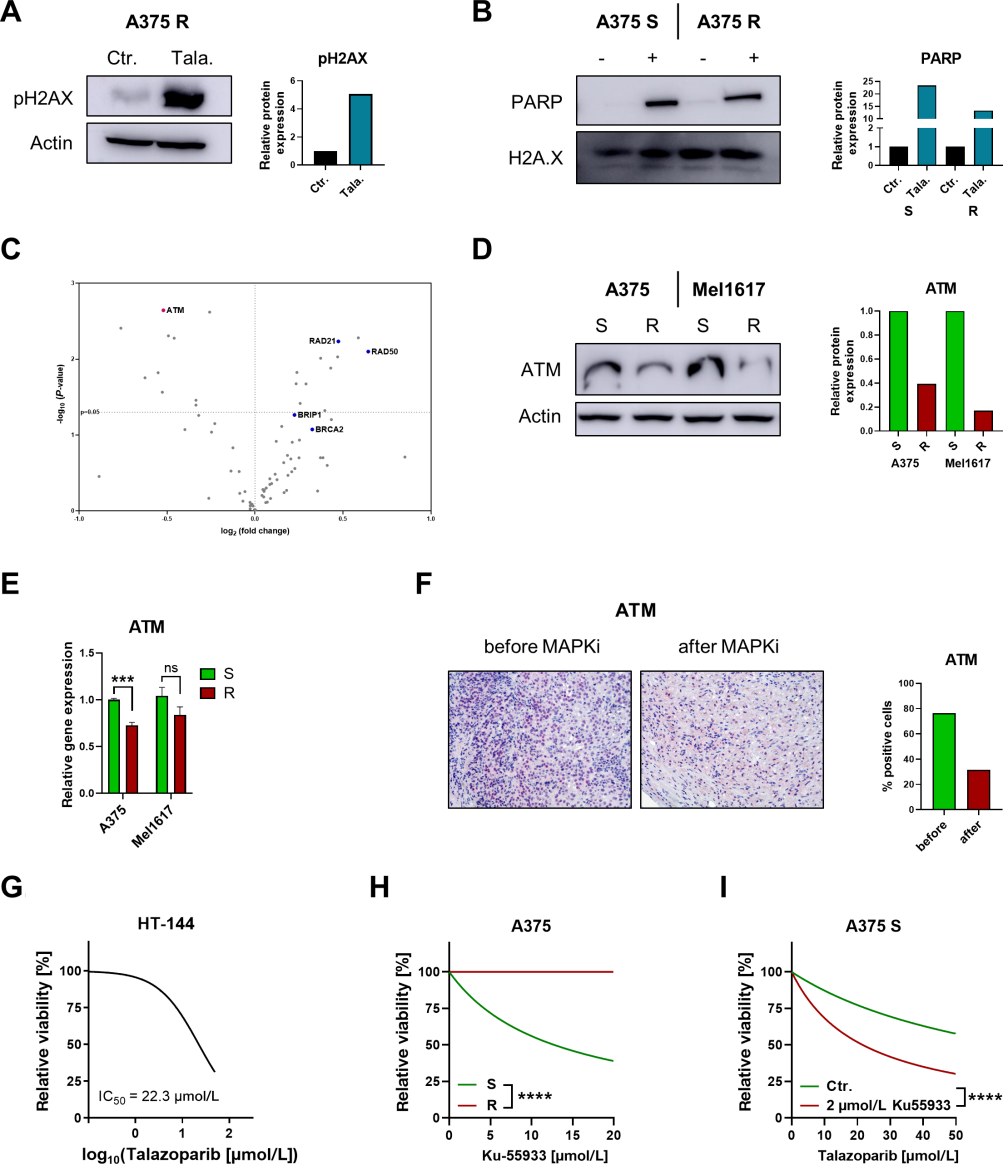
MAPKi resistant cells show decreased ATM expression. **A,** Immunoblot analysis of A375 R cells treated with 5 μmol/L talazoparib (Tala.) for 24 hours or untreated (Ctr.). Relative protein expression is shown. Actin control was reused from Fig. 2E as the Western blots were performed in the same experiment. **B,** Immunoblot analysis of chromatin bound fraction of A375 S and R cells after the treatment with 0.005% MMS and 2 μmol/L talazoparib (+) for 6 hours or untreated cells (−). Relative protein expression is shown. **C,** RT^2^ Profiler PCR Array Human DNA Repair was performed in A375 S versus R**.** log_2_ fold change in the expression of the corresponding genes of R compared with S cells as well as the log_10_*P* values of the respective gene expression changes are shown in the volcano plot. Technical triplicates were performed. **D,** Immunoblot analysis of A375 and Mel1617 S and R cells. Relative protein expression is shown. **E,** Relative ATM gene expression of A375 and Mel1617 S and R is shown. Unpaired *t* test was performed to compare the Ctr. with Tala. group. **F,** IHC of paraffin-embedded tumors of a patient before and after MAPKi resistance stained for ATM. 10X magnification was used. Quantification of the ATM positive cells was performed and % ATM positive cells is shown. **G,** MUH cell viability assay of HT-144 melanoma cell line treated with different concentrations of talazoparib (Tala.) starting at 50 μmol/L for 72 hours. IC_50_ level is shown. **H,** MUH cell viability assay of A375 S and R cells treated with different concentrations of Ku-55933 (Ku.) for 72 hours. Comparison of fits of the nonlinear regression was performed to analyze statistical differences between S and R cells. **I,** MUH cell viability assay of A375 S cells treated with different concentrations of talazoparib for 72 hours. Cells were additionally treated with a stable concentration of 2 μmol/L Ku-55933 or remained untreated. Comparison of fits of the nonlinear regression was performed to analyze statistical differences between cells treated with Ku-55933 or untreated cells.

We found that talazoparib treatment of A375 R cells clearly induced phosphorylation of the histone variant H2A.X (pH2AX) at position serine 139 indicating induction of DNA damage (Fig. 3A). This was also observed in the A375 R tumors of talazoparib-treated mice (Supplementary Fig. S1B). Because the PARP trapping and thereby the induction of DSB is the main mechanism of cellular toxicity of PARPi (24), we tested whether PARPi treatment leads to a higher PARP trapping effect in MAPKi resistant cells. For this, we performed a Western blot analysis, in which we analyzed PARP levels bound to chromatin, and thus, trapped PARP after talazoparib treatment and after short-time treatment with methyl methanesulfonate to induce DNA damage. As expected, higher chromatin bound PARP levels were observed compared with untreated control cells (Fig. 3B). However, no difference between trapped PARP and thus the PARP trapping efficacy in MAPKi resistant cells compared with their sensitive counterparts was seen. Therefore, we proposed that the PARP trapping efficacy is not higher in MAPKi resistant cells compared with their treatment-naïve counterpart. To gain further insight into the high PARPi sensitivity of BRAFi R melanoma cells compared with their treatment naïve, sensitive counterparts, we performed a PCR Profiler Array for human DNA repair genes. Interestingly, we found that in A375 R cells, ATM in particular was downregulated (Fig. 3C; Supplementary Table S1). ATM is a gene important for DNA DSB sensing and for early steps of the DNA damage repair. We confirmed the data by immunoblot analysis (Fig. 3D), qPCR (Fig. 3E), and immunofluorescence staining (Supplementary Fig. S1C) in several matching pairs of S and R melanoma cell lines. The data indicate that RNA and protein expression of ATM is downregulated in BRAFi R cells compared with their sensitive counterparts. Moreover, IHC of matched biopsies from one patient before and after BRAFi resistance development showed a clear downregulation of ATM protein levels in the MAPKi resistant tumor compared with the treatment-naïve tumor (Fig. 3F). Downregulation of ATM in MAPKi resistant melanomas compared with the treatment-naïve tumors was confirmed by database analysis of mRNA sequencing data from matched melanoma samples before and after either BRAFi resistance or BRAFi/MEKi dual resistance development published by Hugo and colleagues (21–23). ATM mRNA levels were significantly reduced in MAPKi resistant melanoma samples compared with samples of the same patient before the onset of MAPKi resistance (Supplementary Fig. S1D). To study the importance of ATM to PARPi treatment in melanoma on a functional level, we used the human melanoma cell line HT-144, that has the homozygous mutation p.W2845* in the ATM gene, resulting in a truncated and thereby inefficient ATM protein. Interestingly, the MUH analysis showed that the ATM-deficient cells are also sensitive to talazoparib treatment (Fig. 3G) and that inhibition of ATM by the ATM inhibitor Ku-55933 is more effective in A375 S cells than the corresponding R cells (Fig. 3H). Most strikingly, downregulation of ATM in A375 S cells via treatment with low concentrations of Ku-55933 sensitized the cells significantly to talazoparib treatment (Fig. 3I). Taken together, these data suggest that BRAFi R cells are more susceptible to PARPi therapy because of lower ATM levels and consequently an inefficient sensing of DNA DSBs.

### Synergistic Killing of Melanoma Cells by a Combination of MAPKi and PARPi

**FIGURE 4:**
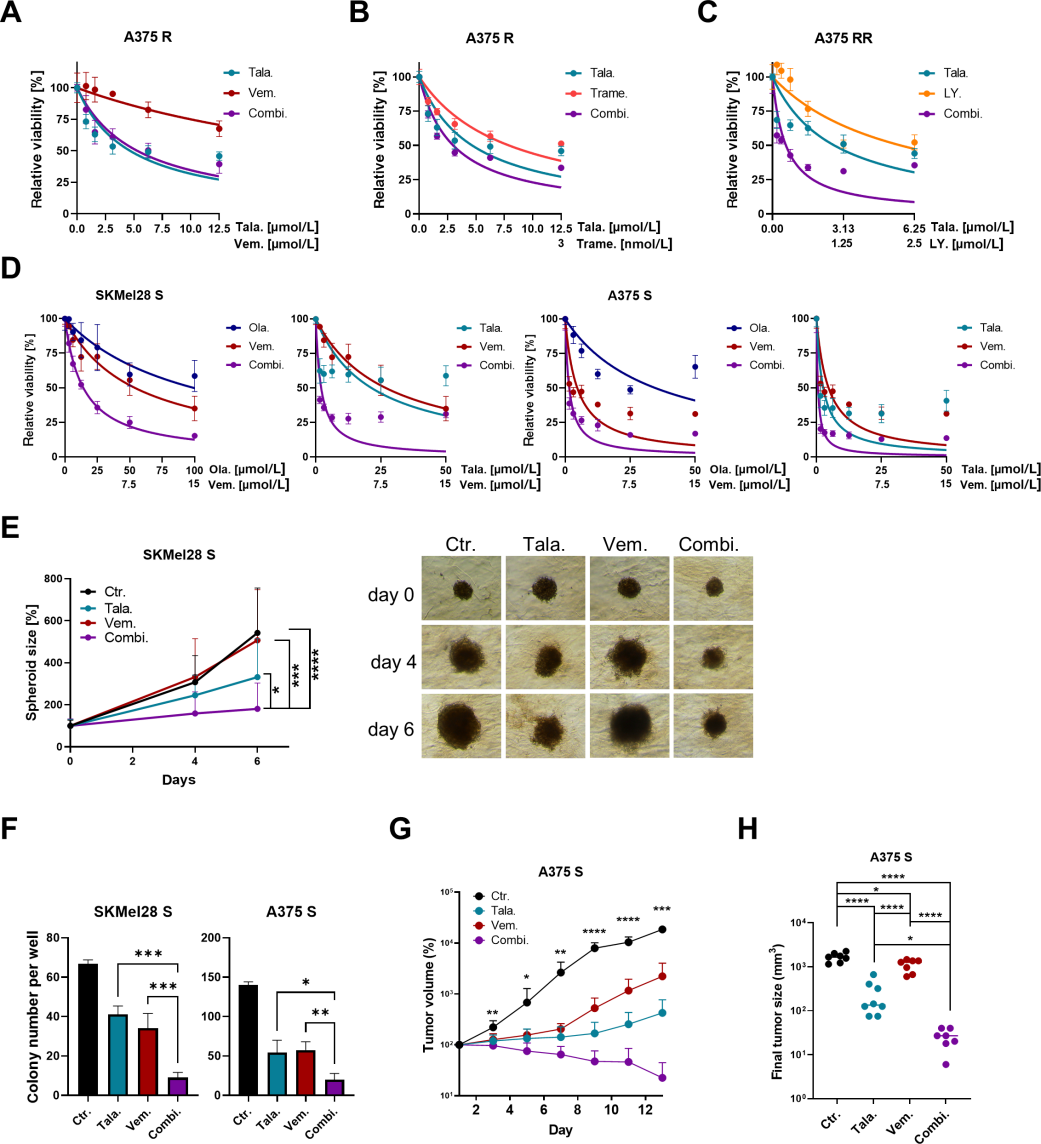
Synthetic lethality elicits synergism in the combination of MAPKi with PARPi. MUH cell viability assay of A375 R or A375 RR cells treated with different concentrations of talazoparib (Tala.), vemurafenib (Vem.; **A**), trametinib (Trame.; **B**), LY3009120 (LY.; **C**) or a combination of these drugs (Combi.) for 72 hours. **D,** MUH cell viability assay of A375 and SKMel28 S cells treated with different concentrations of olaparib (Ola.), talazoparib (Tala.), vemurafenib (Vem.) or a combination of these drugs (Combi.) for 72 hours. **E,** SKMel28 S spheroids were treated with 5 μmol/L talazoparib (Tala.), 2.5 μmol/L vemurafenib (Vem.), a combination of both inhibitors (Combi.) in collagen for 6 days or remained untreated. The total size of the spheroids was quantified and multiple *t* test was performed to compare the groups at day 6. Representative pictures of the spheroids are shown. **F,** Colony formation of SKMel28 S and A375 S was analyzed after the treatment with 2 μmol/L talazoparib (Tala.), 2 μmol/L vemurafenib (Vem.), a combination of both drugs (Combi.) for 7 days or untreated cells (Ctr.). The number of colonies per well was counted. One-way ANOVA was used to compare each group with each other. **G,** NSG mice were treated with control vehicle (Ctr.), 1 mg/kg/day talazoparib (Tala.), 25 mg/kg/day (Vem.), or a combination of both inhibitors (Combi). The xenograft tumor volume in percent compared with treatment start over time is shown. Multiple *t* test was used to compare the Ctr. group with the Combi. group. **H,** The final tumor volume of the individual mice of G is depicted. Multiple *t* test was used to compare the different groups with each other.

Next, we tested whether combined treatment of MAPKi resistant melanoma cells with PARPi and MAPKi increases the effectiveness compared with the monotherapy. Treatment of A375 R cells with talazoparib and the BRAFi vemurafenib did not increase cell death induction compared with talazoparib treatment alone (Fig. 4A; Supplementary Fig. S2A). However, a combination of talazoparib and the MEKi trametinib resulted in a synergistic reduction of melanoma cell viability, as indicated by the low CI values (Fig. 4B; Supplementary Fig. S2A). Consistent with these data, a combination of talazoparib and the panRAF inhibitor (panRAFi) LY3009120 had a synergistic cytotoxic effect on BRAF/MEKi double-resistant A375 melanoma cells (A375 RR; Fig. 4C; Supplementary Fig. S2B). These data indicate that patients with MAPKi resistant melanoma may benefit from a combination of PARPi and MEKi or panRAFi in case of a MEKi resistance.

Next, we asked whether a combination of MAPKi and PARPi would be effective in MAPKi sensitive melanoma cells as a potential additional initial treatment for patients with melanoma. For this, we treated SKMel28 S and A375 S cells either with olaparib, talazoparib, vemurafenib, or the combination of a PARPi and the BRAFi. As shown in Fig. 4D, the combination treatment synergistically reduced the growth of the S melanoma cells (see also Supplementary Fig. S2C and S2D). The different combination treatments led also to a significant lower cytotoxicity in primary human fibroblasts compared with A375 melanoma cells (Supplementary Fig. S2E). The combination treatment was also very efficient in killing melanoma cells cultivated in three dimensions, as it significantly decreased the growth of melanoma cells in spheroids (Fig. 4E). Moreover, the combination treatment significantly reduced the colony formation ability of SKMel28 S and A375 S cells (Fig. 4F). Most strikingly, the combination treatment of vemurafenib and talazoparib completely inhibited growth of A375 S cells *in vivo* (Fig. 4G and H). The tumor volume over time of mice treated with the combination was significantly reduced compared with the tumor volume over time of the solvent-treated control mice. The final tumor size of the mice treated with the combination of BRAFi and PARPi was also significantly lower compared with the tumor size of mice treated solely with vemurafenib or talazoparib. In summary, we demonstrated that a combination of PARPi and MAPKi synergistically reduces the growth of BRAFi sensitive, BRAFi resistant, as well as BRAFi/MEKi double-resistant melanoma cells *in vitro* and *in vivo*.

### Synthetic Lethality Elicits Synergism in the Combination of MAPKi with PARPi

**FIGURE 5:**
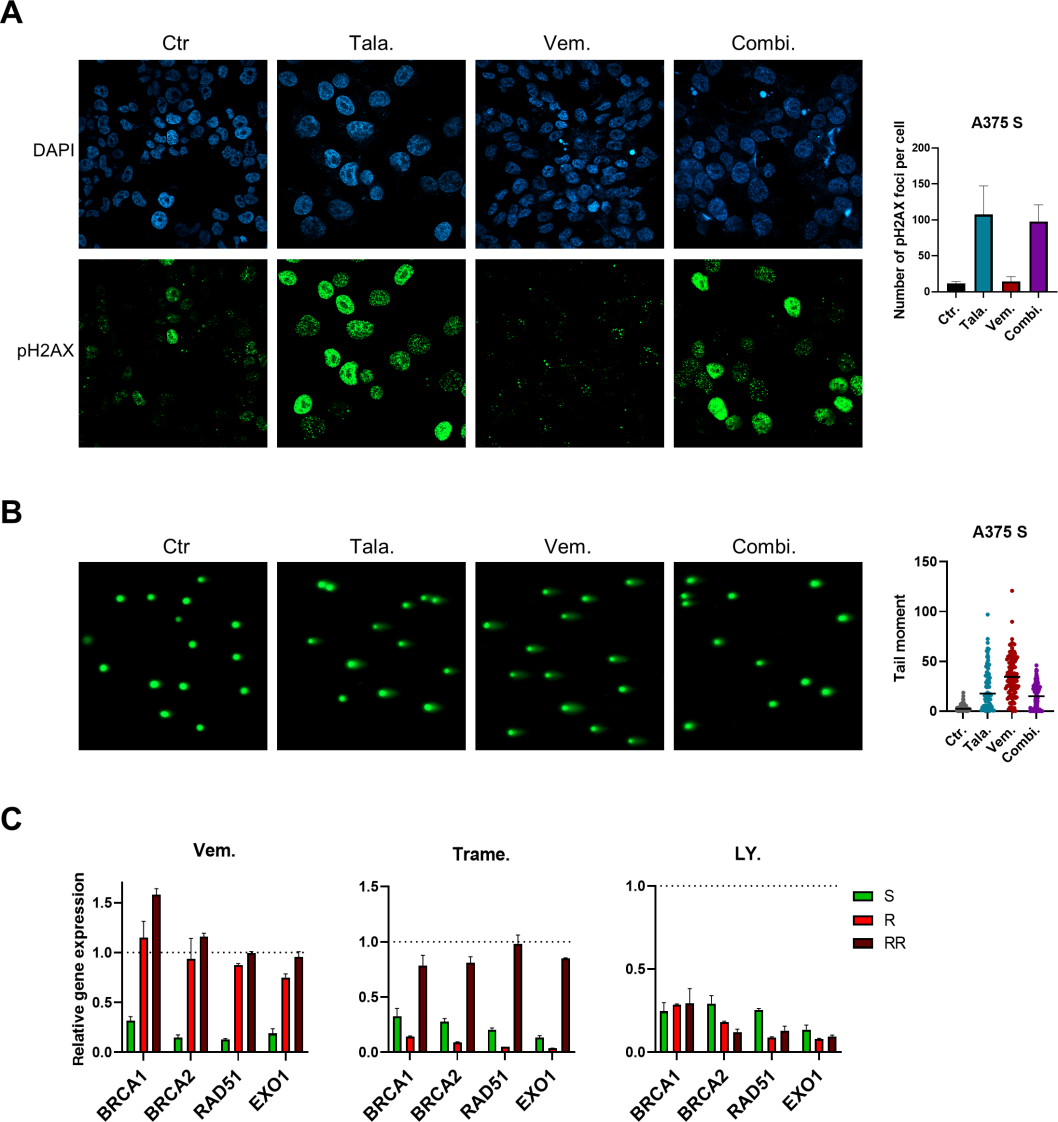
MAPKi induce HRD phenotype and thereby act synthetic lethal in combination with PARPi. **A,** Immunofluorescence of A375 S cells that were treated with 5 μmol/L talazoparib (Tala.), 5 μmol/L vemurafenib (Vem.), or a combination of both inhibitors (Combi.) for 24 hours, or remained untreated (Ctr.). Green: pH2AX, blue: DAPI. 40X magnification was used. Number of pH2AX foci per cell was counted and one-way ANOVA was performed to analyze the differences between the groups. **B,** Comet Assay was performed using A375 S cells that were treated with either 15 μmol/L talazoparib (Tala.), 5 μmol/L vemurafenib (Vem.), a combination of both drugs (Combi.) for 24 hours or remained untreated (Ctr.). Green: DNA. 10X magnification was used. **C,** RNA expression of MAPKi sensitive (S), vemurafenib resistant (R), or vemurafenib and trametinib double-resistant (RR) A375 cells after the treatment with 5 μmol/L vemurafenib (Vem.), 10 nmol/L trametinib (Trame.), or 5 μmol/L LY3009120 (LY.) for 24 hours. The relative gene expression of the treated cells compared with the control group is shown.

To analyze the molecular mechanism of the synergistic effect of PARPi and MAPKi, we determined the extent of DNA damage induction by talazoparib, vemurafenib, or the combination treatment using pH2AX staining of A375 S cells. As shown in Fig. 5A, talazoparib induced DNA damage to a high extent, in contrast to vemurafenib. Interestingly, the combination of talazoparib and vemurafenib did not result in an increase of pH2AX staining extent compared with the single treatments (Fig. 5A). Consistent with that, a comet assay showed no increase in DNA damage of the combined treatment of talazoparib and vemurafenib compared to each single treatment (Fig. 5B). On the basis of these results, we postulated that the synergistic effect of combining PARPi and MAPKi initially does not enhance DNA damage in melanoma cells, but that MAPKi treatment possibly results in the inability to repair the DNA damage induced by PARPi treatment.

Therefore, we analyzed gene expression of the important HRR genes BRCA1, BRCA2, RAD51, and EXO1 after MAPKi treatment in MAPKi S, R, and RR A375 melanoma cells. Interestingly, treatment with the BRAFi vemurafenib, the MEKi trametinib, or the panRAFi LY3009120 resulted in a significant downregulation of these genes in MAPKi sensitive cells, whereas the respective effective MAPKi treatment downregulated these HRR genes in R or RR cells: the MEKi and the panRAFi in R cells and the panRAFi in RR cells (Fig. 5C). To exclude the possibility that the downregulation of HRR genes was solely due to G_1_ cell-cycle arrest induced by vemurafenib, we checked RNA expression of HRR genes in sorted vemurafenib-treated cells that were either in G_1_- or G_2_–M-phase and compared them to the respective untreated cells in G_1_- or G_2_–M-phase. Cells treated with vemurafenib showed decreased HRR gene expression, regardless of cell-cycle phase they were in (Supplementary Fig. S2F). These data indicate that downregulation of the MAPK signaling pathway by the respective MAPKi results in an HRD phenotype in melanoma cells and is therefore synthetically lethal in combination with PARPi.

## Discussion

In this work, we show that PARPi therapy could be an effective treatment option for patients with both MAPKi sensitive as well as MAPKi resistant melanoma as a monotherapy or in combination with MAPKi. We demonstrate that a diverse array of MAPKi induce an HRD phenotype and thus act synthetic lethal in combination with PARPi in MAPKi sensitive and resistant melanoma cells *in vitro* and *in vivo.*

We found lower ATM expression in MAPKi resistant cells and saw higher sensitivity of these cells to PARP inhibitor therapy. Because ATM is known to be an important sensor to activate HRR upon DNA DSBs, our hypothesis is that the higher sensitivity of MAPKi resistant cells to PARPi is explained by lower ATM levels, and thus lower repair of DNA DSBs by HRR. In addition, we found a combination effect between MAPKi and PARPi. This combination effect can be explained by the fact that suppression of the MAPK signaling pathway leads to downregulation of essential HRR genes, such as BRCA1, BRCA2, RAD51, or EXO1. Therefore, BRAF mutant cells treated with MAPK inhibitors are very sensitive to PARP inhibitors due to the downregulation of HRR gene expression. Interestingly, we have been able to detect this combination effect not only in MAPKi sensitive but also in MAPKi resistant cells. We assume that downregulation of ATM decreases the sensing of the DSB, and additional treatment with the MEKi trametinib, or the panRAFi LY3009120 leads to a further decrease in HRR activity, making these cells even more sensitive to PARPi treatment.

Consistent with other publications, we found that cell viability in established melanoma cell lines as well as PDX melanoma cells and PDX melanoma mouse models was clearly reduced by PARPi treatment, especially by talazoparib, a PARPi with high PARP trapping activity (25). We were able to discover that MAPKi resistant melanoma cells are more sensitive to PARPi treatment compared with their treatment-naïve counterparts. Furthermore, we show for the first time that MAPKi resistant melanoma cell lines and patient material have decreased ATM expression, and this confers sensitivity to PARPi treatment. ATM, the most upstream DDR kinase of HRR, acts as a DSB sensor and is recruited and triggered by the MRN complex (26). Activation of ATM results in phosphorylation of crucial proteins, such as BRCA1 and thereby triggers the repair of the DSB via HRR (27). Indeed, several studies have shown that ATM-deficient cancer cells have impaired HRR by inefficient sensing and therefore are particularly susceptible to PARPi treatment (28–30). Also, phase II clinical trials checking the efficacy of PARPi treatment in patients with cancer with among others somatic or germline ATM gene mutations, are currently underway (NCT04042831, NCT03344965, NCT02286687, NCT04030559), even for patients with melanoma (NCT03925350, NCT04633902). Combining our results with previously published data, we hypothesize, that paradoxical hyperactivation of the MAPK signaling pathway, a frequent MAPKi resistance mechanism, upregulates the mTOR signaling pathway, and as a consequence negatively regulates ATM expression (31, 32). Furthermore, downregulation of the TAp73 isoform in MAPKi resistant melanoma cells might have an influence on ATM expression (14). However, we cannot exclude the possibility that other factors associated with MAPKi resistance also have an impact on the increased PARPi sensitivity of MAPKi resistant melanoma cells.

We demonstrated that MAPKi resistant melanoma cells did not respond to inhibition of ATM by Ku-55933, whereas the inhibitor showed marked cytotoxicity in MAPKi sensitive cells. This suggests that downregulation of ATM during the emergence of MAPKi resistance causes MAPKi resistant cells to lose their dependence to ATM signaling compared with MAPKi sensitive cells. This goes in line with a publication showing that ATM-deficient cells do not respond to the ATM inhibitor Ku-55933 (33). Taken together, we conclude that especially the difficult-to-treat patients with melanoma that developed MAPKi treatment resistance may benefit from PARPi treatment, and that ATM might be a promising biomarker for treatment response.

We found that PARPi treatment significantly decreased the migratory and invasive potential of melanoma cells. Further publications support our data by showing that PARPi lead to suppression of migration, invasion, and colonization of distal organs of metastatic melanoma cells (34, 35). These findings suggest that PARPi treatment could not only lead to a shrinkage of the primary tumor, but also reduce the metastatic potential of melanoma cells. In agreement with other studies, we could show that DNA damage induction by PARPi treatment of melanoma cells leads to clear G_2_–M-phase arrest and subsequent induction of apoptosis (25, 35). Cell death was mainly mediated by p53-associated activation of proapoptotic genes and massive induction of p21. p53-dependent p21 activation is known to be crucial for G_2_–M checkpoint arrest following DNA damage (36–38). This eventually led to mitotic catastrophe and apoptosis, resulting in cancer cell death (39, 40).

Numerous preclinical and clinical studies have been looking at the use of PARPi in combination with chemotherapy, ionizing radiation, or immunotherapies (8, 41, 42). However, the potential of combining PARPi with targeted therapies, especially with MAPKi, is still unknown. Previously, another group and ours were able to decipher that suppressing the MAPK pathway downregulates HRR gene expression in MAPKi sensitive melanoma cells via the transcription factor ELK1, but the HRR regulation in MAPKi resistant cells remains unknown (11, 12). Therefore, we used a MEKi to suppress the MAPK signaling pathway in BRAFi resistant melanoma cell lines, and a panRAFi in BRAFi/MEKi double-resistant melanoma cells, that has proven to suppress the MAPK signaling pathway in BRAFi/MEKi double-resistant cancers (43, 44). We were able to show that downregulation of the MAPK signaling pathway specifically induces an HRD phenotype by suppressing the important HRR genes BRCA1, BRCA2, RAD51, and EXO1. Because the MAPK signaling pathway is mainly responsible for cell growth, we assume that an activation of it, and thus a faster cell division also requires a higher HRR activity, and therefore the HRR genes are regulated via the MAPK signaling pathway (45).

Most strikingly and in line with other publications, we demonstrated that PARPi in combination with MAPKi show a synergistic cytotoxicity in melanoma cells both *in vitro* and *in vivo* (12, 13). MAPKi results in an HRD phenotype and the combination with PARPi leads to a synergistic synthetic lethal interaction of both therapies. Taken together, we propose that patients with treatment-naïve BRAF-mutated melanoma would benefit from a combination treatment with BRAFi and PARPi as resistance quickly develops in the majority of MAPKi single treated patients. BRAFi/MEKi pretreated and thereby double-resistant patients as well as MEKi single resistant patients may benefit from a combination therapy of panRAFi and PARPi. However, we found that the combination of talazoparib with vemurafenib has significantly higher toxicity *in vitro* on primary human fibroblasts as the MEKi or panRAFi. Therefore, a potential toxicity may limit the clinical translation of the combination therapy. To mitigate a potential toxicity of the combination treatment, we propose to use in clinical studies talazoparib in combination with MEKi or panRAFi. Consistent with this, preliminary data from a phase I clinical trial in which solid tumors with Ras pathway alterations were treated with the PARPi olaparib and the MEKi selumetinib showed no grade 4 adverse events and were generally well tolerated (46).

With this project, we support the concept that not only BRCA-mutated patients benefit from PARPi therapy, but also non–BRCA1-mutant tumors that have BRCAness and thus a HRD phenotype may be treated with and benefit from PARPi treatment (47–49). There are currently numerous clinical trials underway focusing on the efficacy of PARPi therapy on HRD cancers, including patients with melanoma (NCT03207347, NCT03925350, NCT04633902, NCT05482074, NCT04187833, NCT05169437). Olaparib has even been approved for patients with HRD prostate cancer with a mutation in at least one of 14 HRR-related genes (50). However, these involve patients that carry a mutation in one of the HRR genes. Our data are consistent with the outcome of another study indicating that patients do not necessarily have to have a mutation in the HRR pathway to respond well to PARPi treatment, but that suppression of the MAPK pathway with targeted therapy leads to reduced HRR gene expression inducing an HRD phenotype (12).

In summary, our study indicates that PARPi should not be limited to the treatment of patients with somatic or germline mutations in the HRR pathway, but that a combined treatment of melanoma patients with PARPi and MAPKi induces synthetic lethality by inducing a HRD phenotype and stops tumor growth.

## Supplementary Material

Supplementary Figure 1 PARPi treatment in melanoma cells

Supplementary Figure 2 Synergistic interaction of PARPi and MAPKi treatment

Supplementary Table 1 RT2 ProfilerTM PCR Array Human p53 Signaling Pathway and Human DNA Repair Pathway
