# Supplementary material for: PARP Inhibitors Effectively Reduce MAPK Inhibitor Resistant Melanoma Cell Growth and Synergize with MAPK Inhibitors through a Synthetic Lethal Interaction In Vitro and In Vivo
Source: Cancer Res Commun. 2023 Sep 5;3(9):1743–55. doi: 10.1158/2767-9764.CRC-23-0101 (PMC10478790; doi:10.1158/2767-9764.CRC-23-0101)
Supplement: Supplementary Figure 1 — PARPi treatment in melanoma cells [file crc-23-0101-s01.pdf]

Supplementary Figure S1:

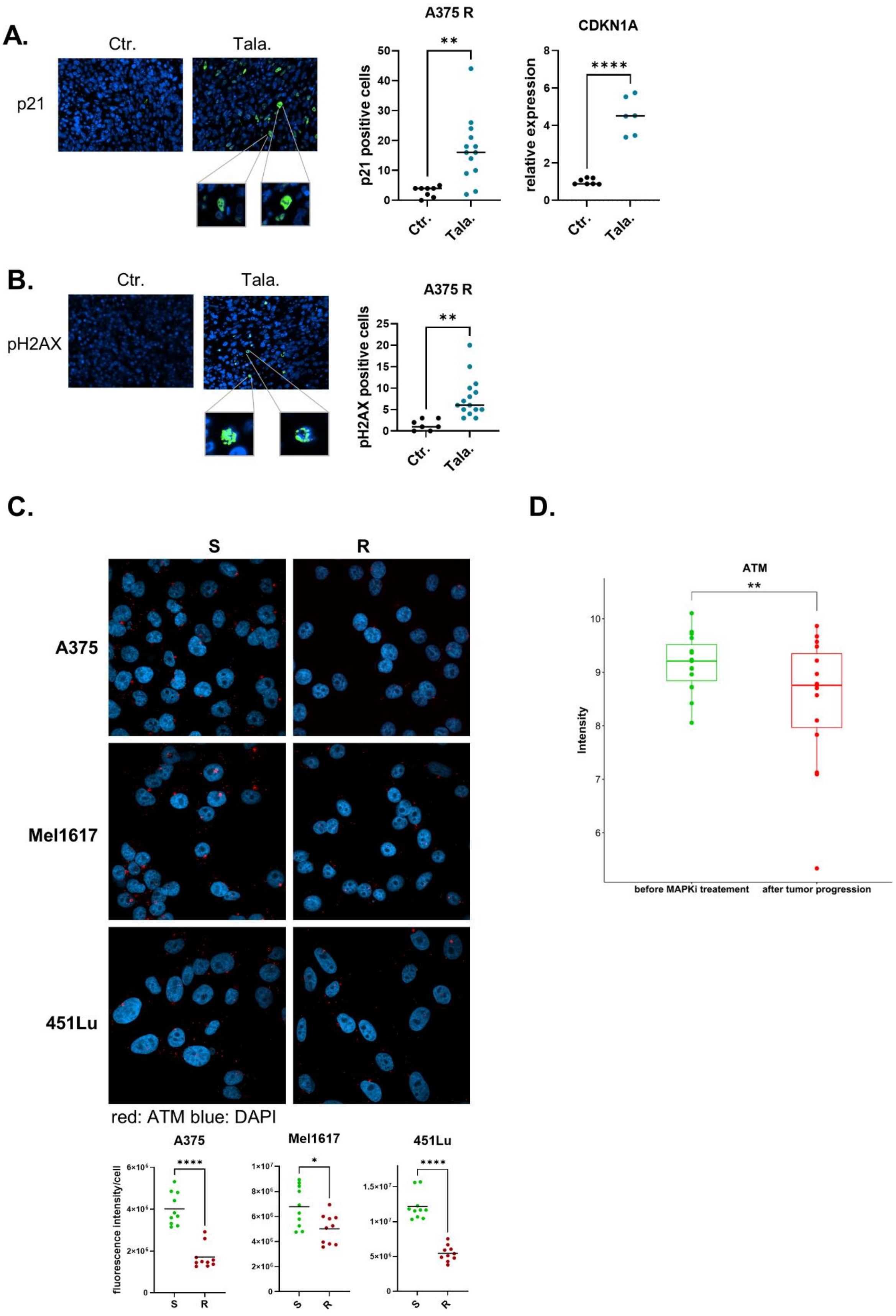

Supplementary Figure S1: PARPi treatment in melanoma cells.

A.-B. Immunofluorescence of p21 (**A.**) and pH2AX (**B.**) of the mouse tumors from Figure 1D. Green: p21/pH2AX. Blue: DAPI. Number of p21/pH2AX positive cells per image section is shown. 20x magnification was used. **C.** Immunofluorescence of ATM in 451Lu, A375, and Mel1617 S and R cells was performed. Red: ATM, blue: Hoechst. 40X magnification was used. 10 images with on average 25 cells per image were analysed. The fluorescence intensity per cell is shown. **D.** Normalized ATM RNA expression levels of melanoma patients before MAPKi treatment and after tumor progression. The data were obtained from the databases GSE50509 and GSE61992.
