# Supplementary material for: PARP Inhibitors Effectively Reduce MAPK Inhibitor Resistant Melanoma Cell Growth and Synergize with MAPK Inhibitors through a Synthetic Lethal Interaction In Vitro and In Vivo
Source: Cancer Res Commun. 2023 Sep 5;3(9):1743–55. doi: 10.1158/2767-9764.CRC-23-0101 (PMC10478790; doi:10.1158/2767-9764.CRC-23-0101)
Supplement: Supplementary Figure 2 — Synergistic interaction of PARPi and MAPKi treatment [file crc-23-0101-s02.pdf]

Supplementary Figure S2:

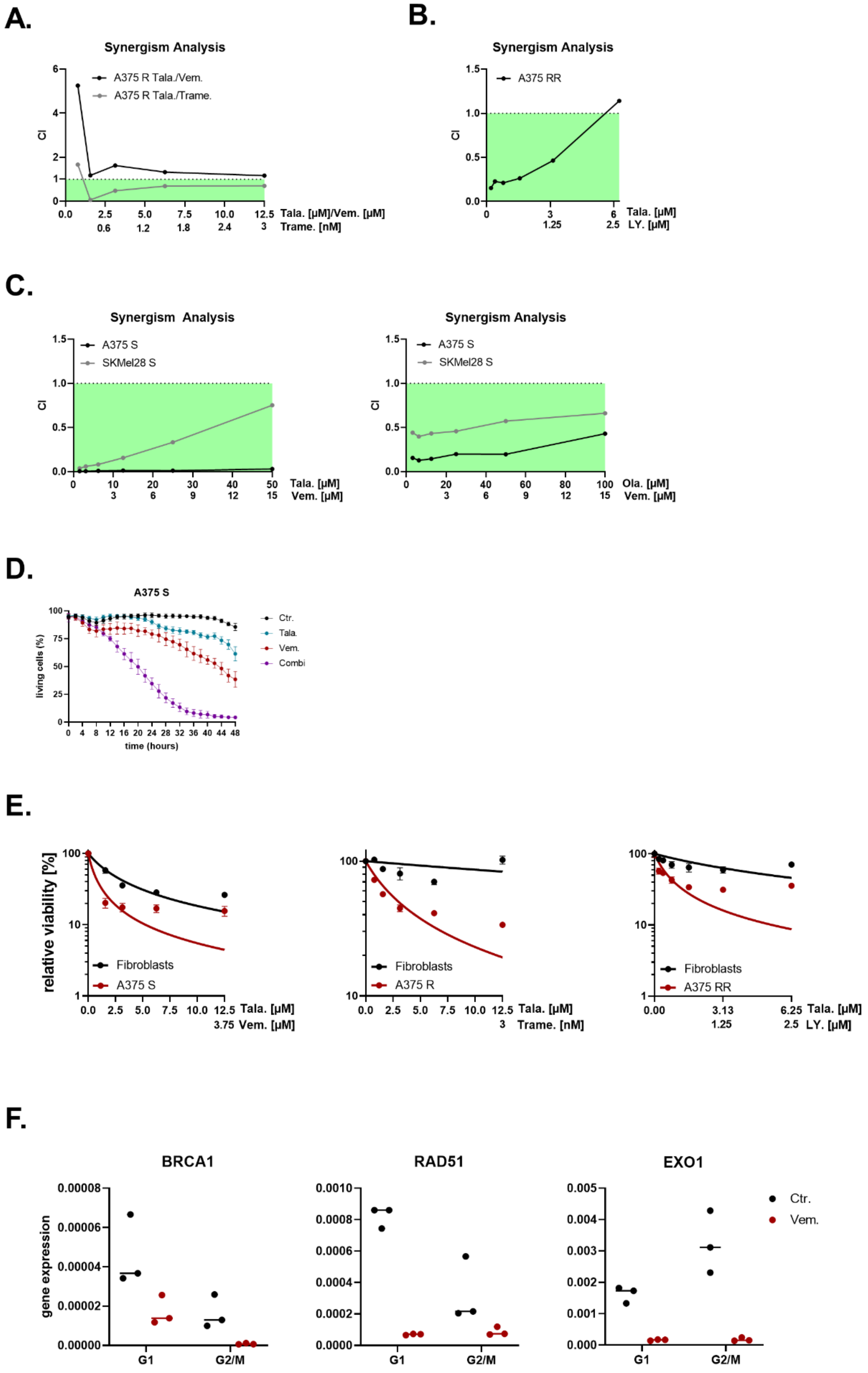

Supplementary Figure S2: Synergistic interaction of PARPi and MAPKi treatment.

A.-C. Synergism analysis was performed using the data of Figure 4A (**A.**), Figure 4B (**A.**), Figure 4C (**B.**), and Figure 4D (**C.**) treated with combinations of olaparib (Ola.), talazoparib (Tala.), vemurafenib (Vem.), trametinib (Trame.), or LY3009120 (LY.) for 72. Synergism analysis was analyzed using the combosyn software. The calculated combined index (CI) values reflect the degree of the combined treatment with: synergistic effect = 0<CI<1, and additive effect = CI≥1 **D.** A375 S cells were treated with 5 µM talazoparib (Tala.), 5 µM vemurafenib (Vem.), or a combination of both drugs (Combi.) for 48h or remained untreated (Ctr.). Every 2 hours pictures of the cells were taken with the Incucyte SX1 system. Incucyte advanced label-free classification analysis software was used to calculate the percent of living cells. **E.** MUH cell viability assay of primary human fibroblasts treated with different concentrations of the PARPi talazoparib (Tala.) and the BRAFi vemurafenib (Vem.), the MEKi trametinib (Trame.), or the panRAFi LY3009120 (LY.) for 72h. A375 melanoma cells (S for Tala.+Vem., R for Tala.+Trame, and RR for Tala.+LY.) from Figure 4A-D were used for comparison. **F.** A375 S cells were sorted in the different cell cycle phases. RNA expression of either in G1 or in G2/M phase treated with 5 µM vemurafenib (Vem.) or untreated (Ctr.).
